# Microwave processing: A boon for oral pathologists

**DOI:** 10.4103/0973-029X.80031

**Published:** 2011

**Authors:** Prasad G Kango, RS Deshmukh

**Affiliations:** *Department of Oral and Maxillofacial Pathology and Microbiology, Bharati Vidyapeeth Dental College and Hospital, Pune, India*

**Keywords:** Microwave, tissue processing, oral tissues

## Abstract

**Background:**

Research in oral and maxillofacial pathology has unlimited potential. We use every technology available to us for better and faster reliable diagnosis. But in most institutions, private laboratories and multispecialty hospitals, tissue processing takes considerable time, and therefore delays the diagnosis, which is required in urgent cases. We, in this institution, conducted a study to hasten the processing by using a simple kitchen microwave.

**Aim:**

To analyze tissue sections processed by microwave as compared to the gold standard of conventional processing.

**Settings and design:**

Studies published from 1970 till 2008, used body tissues such as brain, liver, kidney, heart, and lungs, for microwave processing. Oral tissues were not processed in microwave till now, except one study by Dr Shivaparthasundaram *et al*., in 2008. This is the second such study that used a sample size of 50 cases.

**Materials and Methods:**

A kitchen microwave was used for irradiation of the tissues. Conventional processing was carried out as per departmental protocol. A total of 50 microwave-coded slides were mixed with 50 conventional slides. All 100 slides were evaluated by four different pathologists.

**Statistical analysis:**

The result was subjected to statistical analysis using Chi-square test.

**Result and Conclusion:**

It was found that to make a diagnosis, microwave-processed tissue were at par with the conventional technique. Thus, it is time to move on from conventional processing to microwave processing to yield faster and reliable results.

## INTRODUCTION

Conventional tissue processing is as old as 100 years and still remains the gold standard against which all new technologies and methods need to be assessed. A laborious and tedious manual sequence, tissue processing is of paramount importance for having good thin sections without artifacts.

Microwaves, which were invented by Percy Spencer in 1945, created a small buzz and soon became an integral part of our daily lives.[1] Although widely used in food processing, chemical, pharmaceutical, and many other industries for many years, it was Kok and Boon from The Netherlands and Anthony Leong from Australia who advocated microwave heating for fixation and processing of tissues in the late 1980s. Thus, a novel histoprocessing method for paraffin section was developed and fast processing was possible due to stimulated diffusion of the heated reagents.

This study uses microwaves for the processing of oral tissues and Analysis the cellular and nuclear morphology as well as staining characteristics. Comparison is done with the conventional technique with the same parameters.

### Aim

To evaluate the quality of stained sections by both techniques.

### Objective

Microwave ovens can generate heat from within, and thereby warm tissues and reagents uniformly in a short time. The objective of this study is to process the tissues in a microwave oven and then compare the sections with the gold standard of conventional processing.

## MATERIALS AND METHODS

National microwave oven (Matsushita electric industrial Co Ltd, Made in Japan); Model no.: NN – 5208; Serial no.: N 09130085; Input- 1180 W, 5.2 A, 240 V, 50 Hz; Output- 600 W, 2450 MHz.

- Jar- 600 ml; two jars of 200 ml each; seven glass vessels of 200 ml each
- 10% Formalin; acetone; absolute alcohol; xylene; chloroform and paraffin wax

### a) Sample selection

Specimens for this study were selected randomly from those received in the department of Oral and Maxillofacial Pathology, Bharati Vidyapeeth Dental College and Hospital, Pune, and from private hospitals and clinics in and around Pune.

1. Only soft tissue was preferred for this study
2. The sample size was 1 cm × 1 cm or greater
3. The thickness of 5-8 mm was taken for microwave processing
4. The tissue was then divided into equal halves; one was processed in a microwave and the other was processed conventionally.

### b) Fixation of samples

10% formalin was used as a fixative.

### c) Conventional tissue processing

The processing was started at 8.30 am; the cassette was kept in water for 1 hour to remove formalin. Dehydration in acetone was done in four steps of 1 hour each, using graded acetone (70%, 80%, 90%, and 100%) from 9.30 to 1.30 pm. Tissue was cleared in xylene in three steps of 1 hour each (ie, 1.30 pm to 4.30 pm), using 100% concentration of the solution. At the end of the college hours, the cassette was kept in paraffin wax bath I for impregnation, which had a preset temperature of 40°C. The subsequent morning at 9.00 am, the tissues were removed from paraffin wax bath I and placed in wax bath II for 1 hour (preset temperature of 40°C). This was done to allow proper impregnation of the wax into the tissues. At 10.00 am, tissue was removed from the wax bath II and embedded. This procedure was followed routinely.

All processing was done at room temperature, except for impregnation and embedding.

### d) Microwave tissue processing

#### 1. Standardization of the procedure

Xylene has a high boiling point and low microwavability, ie, it will take double the time for the same amount of reagent to get heated. Therefore, in this study, chloroform was used as a clearing agent for microwave tissue processing.

A pilot study was done in which each reagent was microwaved for 5 and 10 minutes. The tissues microwaved for 5 minutes showed improper dehydration, clearing, and impregnation, while those microwaved for 10 minutes showed improper clearing and tissue breakage while cutting. The tissues and reagents were microwaved for 15 minutes. The dehydration, clearing, and impregnation of tissues were found to be satisfactory. Hence, this method was taken as a standard protocol for all tissues.

The time taken for dehydration, clearing, and impregnation was same as that of Klump[2] *et al*., where the following steps were used:

- Absolute alcohol: 15 minutes (one step)
- Chloroform: 15 minutes (“)
- Paraffin impregnation: 15 minutes (“)

The solutions are not covered with the lid because we had two jars: the first jar contained a 200 ml solution (alcohol or chloroform) along with the tissue inside, and the second one contained a water load of 500 ml and placed next to the first jar. In this way, We were able to control the excess heat, which was absorbed by the water.

Due to very small size of the oral tissues, the temperature of the tissue cannot be measured. Thus, it was assumed to be similar to that reached in the solution, which was in the range of 45°C-58°C.[3]

The power of the microwave available for this model was warm, low, medium low, high. In this study, the power modes used were as follows:

Power mode for each solution:

- First 5 min- low mode and next 10 min - warm mode
- Power mode for paraffin wax:
- First 5 min- medium low mode and next 10 min - low mode
- This was done to allow chloroform to effectively boil out of the tissue and replace it with paraffin.

#### 2. Embedding, section cutting, and staining were done simultaneously for both blocks

#### 3. Studying of sections

A total of 50 pairs of slides were obtained; one each for microwave and conventional tissue processing. All 100 slides were coded by an independent observer, in which 50 slides were coded with an alphabet (A) and the other 50 slides were coded (B). Four observers evaluated all 100 slides without the knowledge of the type of processing used.

### Criteria for evaluation of quality of sections

- For cellular morphology evaluation, greater eosinophilia of cytoplasm producing enhancement of the nuclear-cytoplasmic contrast, good stroma, whether secretory products are appreciable, red cell lysis absent, whether differentiation can be made between inflammatory cells.[4] If most features were present, then it was called distinct and if there was granularity of cytoplasm,[4] focal condensation of stroma, cellular outline blurred,[5] mucin not seen, red blood cells lysed (focal or generalized),[4] and no differentiation could be made between inflammatory cells then it was classified as indistinct.
- Evaluation of slides for nuclear morphology was done on the basis of chromatin condensation, prominent nuclear membrane, and crisp staining of the nucleus and mitotic activity, if appreciable.[4] It was distinct if all features were appreciated, and indistinct if smudging and pyknosis of nuclei were seen.[6]
- Staining of tissues was evaluated as poor, satisfactory, and good. Poor indicates that the tissue failed to take up stain adequately, stained unevenly or had artifacts in processing or staining. Satisfactory indicates that details were not visualized up to the mark, but slide was suitable to give diagnosis. Good means good contrast between the nucleus and cytoplasm, and visibility of details along with brilliance of staining.[4,6,7] The overall architecture of the epithelial tissue and connective tissue was assessed as per the above-mentioned criteria.[6]

After evaluation by all four observers, the processing code was broken and results were subjected to statistical analysis.

## RESULTS

The reliability test was done to evaluate the interobserver variations. Table 1 shows that all alpha values obtained were statistically significant. Thus, all observers were assumed to be reliable. For further analysis, one of the observers was randomly selected, who was observer number 2. The alpha values in percentages, where in all observers were 75% in concordance for cellular morphology, 60% for nuclear morphology, and 56% for staining quality.

**Table 1:** Reliability test was carried out among all four observers to evaluate the alpha value

Parameters	Alpha value	Percentage
Cellular morphology	0.7548	75
Nuclear morphology	0.6064	60
Staining characteristics	0.5650	56

**Figure 1:**
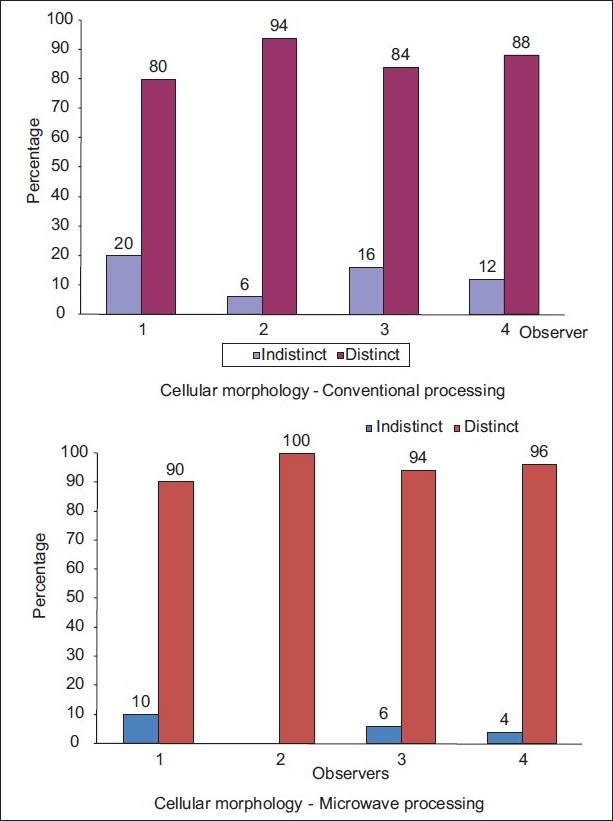
Comparison between microwave and conventional tissue processing for cellular morphology

Table 2 shows the histopathological evaluation of the slides processed by microwave and conventional technique. The Pearson Chi-square test was done and the analysis showed that both techniques were showing the same or similar cellular morphology. The results were statistically insignificant. Figure 1 shows that all four observers had a similar finding for cellular architecture, which was distinct in both conventional and microwave processed tissue, whereas the indistinct cellular morphology was seen more in conventional processing. This indicates that microwave processing did not affect the tissue architecture in any way.

**Table 2:** Histopathological evaluation for cellular morphology

Cellular morphology	Grades	Technique		Total
		Microwave	Conventional	
Indistinct	0	0	3	3
Distinct	1	50	47	97
Total		50	50	100

Pearson Chi-square test; Value- 3.093; P value- 0.079; Statistically insignificant

**Figure 2:**
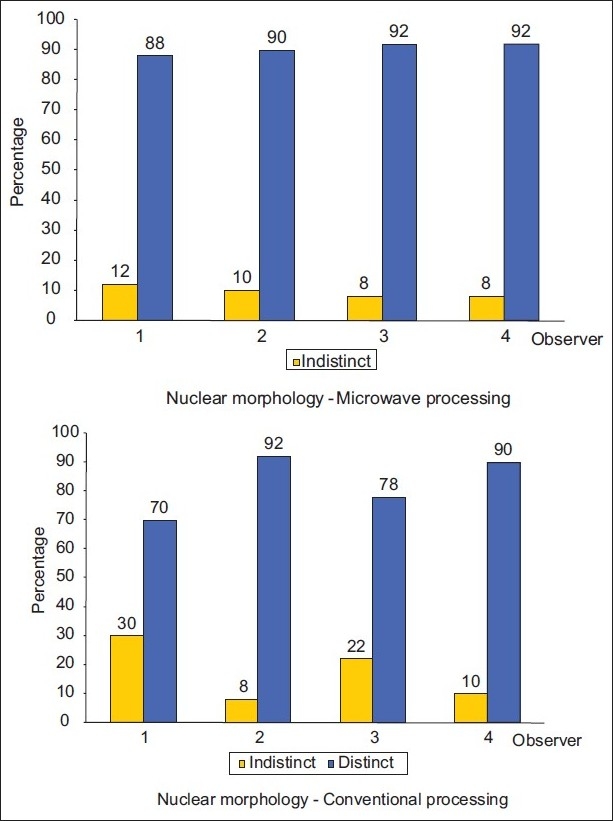
Showing comparison between microwave and conventional tissue processing for nuclear morphology

**Figure 3:**
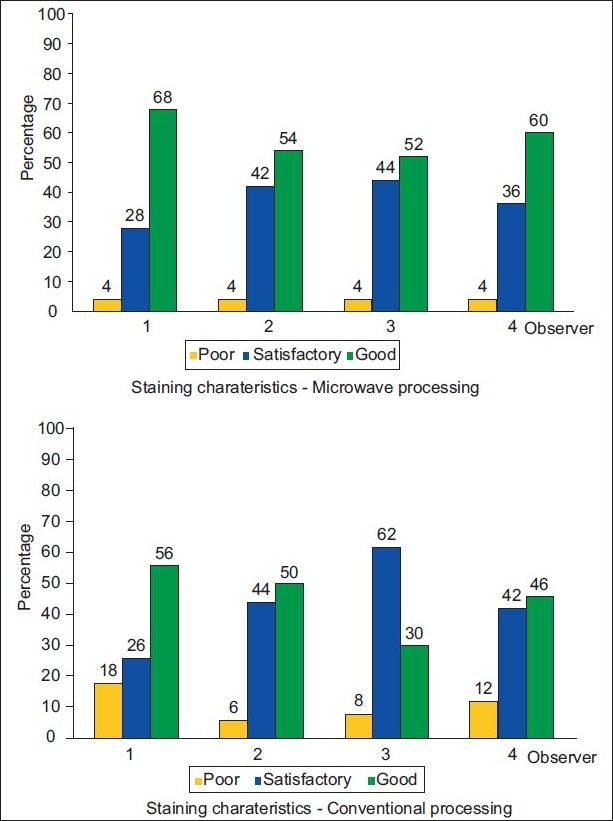
Showing staining characteristics of microwave and conventional tissue processing technique

Similarly, Table 3 shows nuclear characteristics in which the nuclear chromatin, nuclear membrane and prominent nucleoli were evaluated. Barring few slides, the results were statistically insignificant, thus proving that microwave tissue processing produces similar or better results. Figure 2 shows that nuclear morphology was distinct in both techniques. Table 4 shows the staining characteristics of the slides. Grading of 0 was taken as poor where there was uneven staining and the nucleus or cytoplasm was not discernable. While grade 1 was given for satisfactory staining, this was seen in many slides of microwave and conventional tissue processing as seen in Figure 3. Those slides which had excellent nuclear and cytoplasmic contrast were rated as good and the grade given was 2. Microwave processed tissue sections had good cytoplasmic contrast as compared to those processed by the conventional technique.

**Table 3:** Histopathological evaluation for nuclear morphology

Nuclear morphology	Grades	Technique		Total
		Microwave	Conventional	
Indistinct	0	5	4	9
Distinct	1	45	46	91
Total		50	50	100

Pearson Chi-square test; Value- 0.122; *P* value- 0.727; Statistically insignificant

**Table 4:** Histopathological evaluation for staining characteristics

Staining characteristics	Grades	Technique		Total
		Microwave	Conventional	
Poor	0	2	3	5
Satisfactory	1	21	22	43
Good	2	27	25	52
Total		50	50	100

Pearson Chi-square test; Value- 0.300; *P* value- 0.861; Statistically insignificant

Table 4 shows that there were only few sections in both conventional and microwave tissue processing that showed poor staining characteristics. Thus, the result is statistically insignificant.

The histological assessment was done by three independent observers and myself. The observers were as follows: observer no 1- Oral pathologist with 15 years of teaching experience; observer no 2- a General pathologist with a teaching experience of 7 years; and observer no 3- an Oral pathologist with a teaching experience of 2 years.

The microscopic quality of the sections was comparable to, or slightly better than, conventionally processed tissue having the same formalin fixation time. The architecture in the sections was well preserved as in concordance with Kok *et al*.[8]

From these results, we believe that rapid microwave-assisted tissue processing is the optimal method for producing quality sections. Also, excellent microscopic sections obtained by this technique revealed no differences in the cellular and nuclear morphology in several types of tissues.

## DISCUSSION

The formula for diffusion states that: the average squared distance covered by a particle in solution is proportional to the diffusion time. This shows that the thickness of biopsies should be small: three times as thick means nine times as long for comparable effects. It should be noted that the length and breadth does not matter here.[9]

Proteins in the tissue are denatured by absolute alcohol to such a degree that subsequent heating does not have any additional influence on the light microscopic results. Alcohol is also used as a coagulant fixative that hardens the tissue, and this is needed for cutting of sections. This hardening effect is caused by coagulation of proteins.[7]

The literature on microwaves for histoprocessing comprises several papers that advocate the use of domestic microwave ovens. In the book by Kok and Boon, the total processing time was 111 minutes when 500-ml containers were used and 30 blocks were prepared. In all steps, the working temperature of 75°C was maintained.[10]

In this study, the microwave processed tissue sections had better cytoplasmic [Figure 1] and nuclear details [Figure 2], with good erythrocyte integrity and lymphocyte appearance than the conventional method [Tables 2–4]. Overall, the quality of microscopic tissues from conventional and microwave processing methods were identical. It was not possible to distinguish between the two techniques by studying the tissue section as seen in studies conducted by Morales *et al*.,[11,12] Mathai AK *et al*.[13]

Boon *et al*.,[7] Chaudhari *et al*.,[5] and Morales *et al*.,[11,12] found the tissue architecture, stroma, secretory products, cell and nuclear morphology were same between conventionally processed and microwave processed tissue, which was also seen in this study. The tissue architecture was well maintained with no shrinking or spongy pattern [Figure 1]. No crisp ethyl alcohol patterns of nuclear features were seen as observed by Boon *et al*.[7]

In this study, the effect of microwaves on the different types of tissue such as epithelium, fibrous, and glandular, showed no statistically significant variation, as also seen in studies by Panja *et al*.[14]

**Figure 4:**
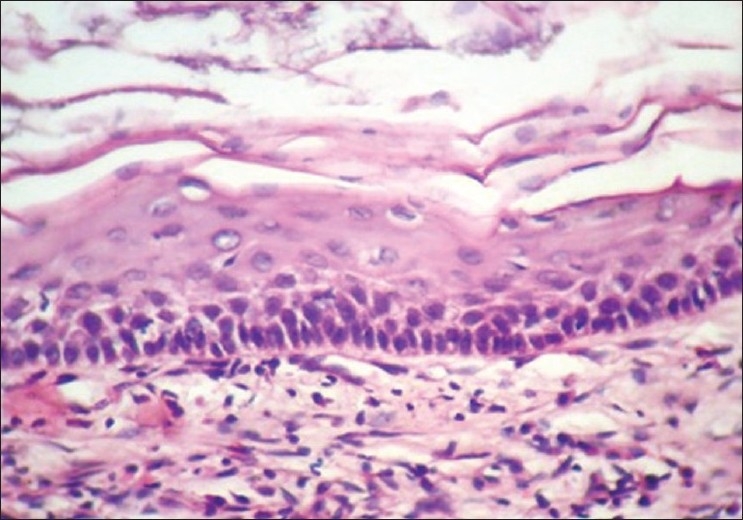
Odontogenic Keratocyst. 40× Microwave processed

**Figure 5:**
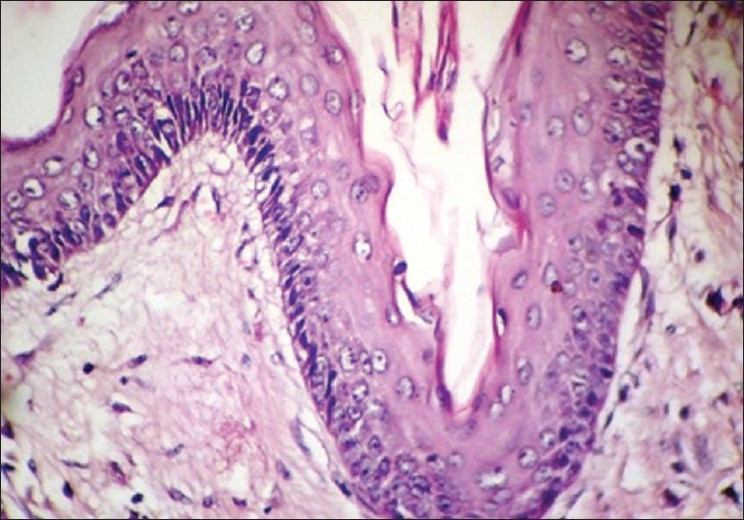
Odontogenic Keratocyst. 40× Conventional processed

Boon *et al*.[7] found that in microwave processed tissues the epithelium was of better quality, while the stroma had a slightly different appearance, in that it appeared to be slightly more condensed focally. Similar results were seen in this study where the epithelium showed excellent nuclear and cytoplasm contrast [Figures 4 and 5], and the intercellular bridges were also appreciable. Focal condensation of connective tissue is of no importance in diagnostic pathology, as explained by Kok.[9]

**Figure 6:**
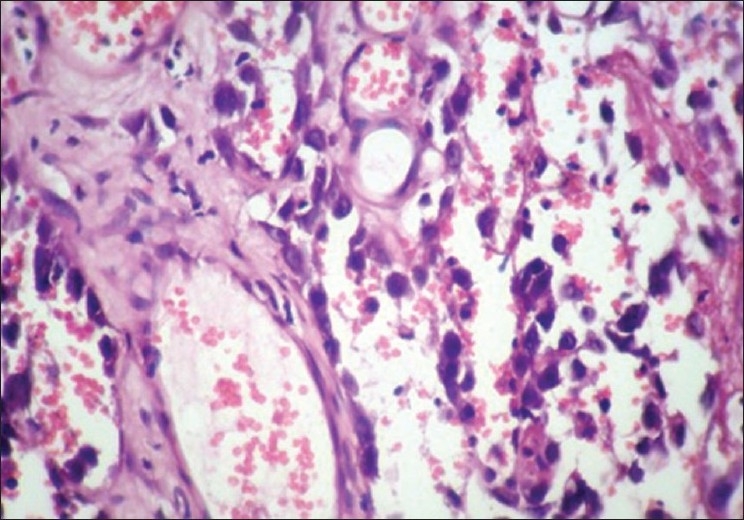
Angiosarcoma. 10× Microwave processed

Red cells were not lysed [Figure 6] by microwave treatment in this study, whereas in studies by Hopwood *et al*.,[15] Mayers,[16] Leong,[4] and Bernard,[17] the red cells were lysed. Inflammatory cells such as plasma cells and lymphocytes were distinguishable from each other [Figure 4], and this was also seen in studies by Hopewood *et al*.[15]

**Figure 7:**
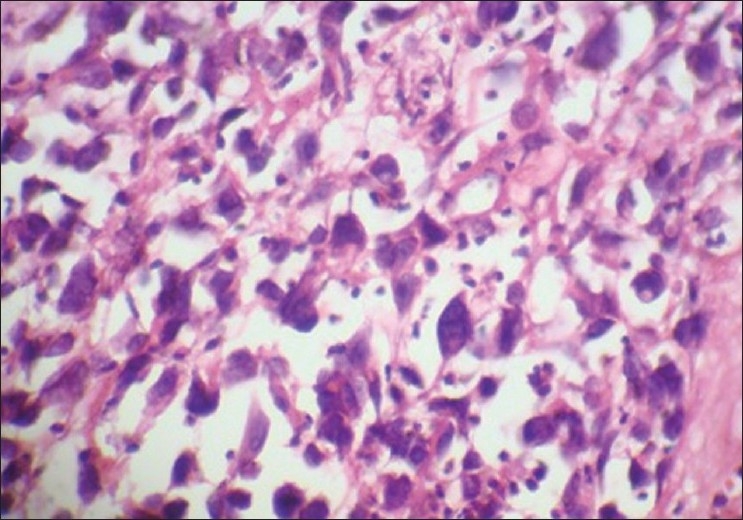
Angiosarcoma. 10× Conventional processed

**Figure 8:**
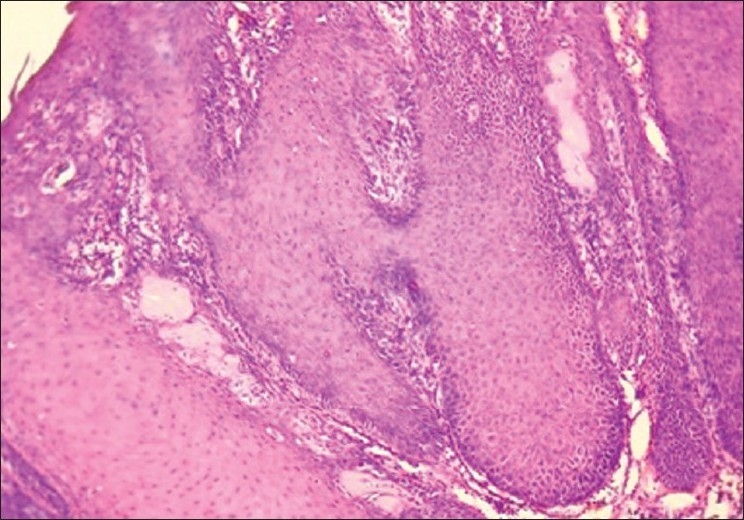
Verrucous carcinoma. 10× Microwave processed

**Figure 9:**
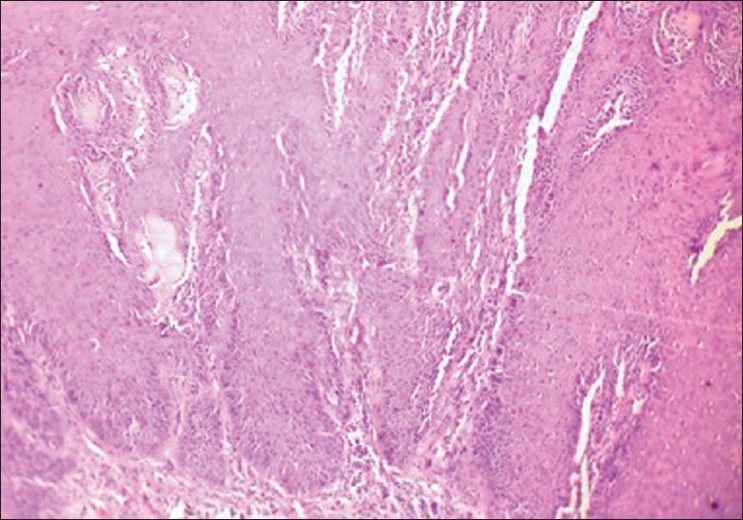
Verrucous carcinoma. 10× Conventional processed

**Figure 10:**
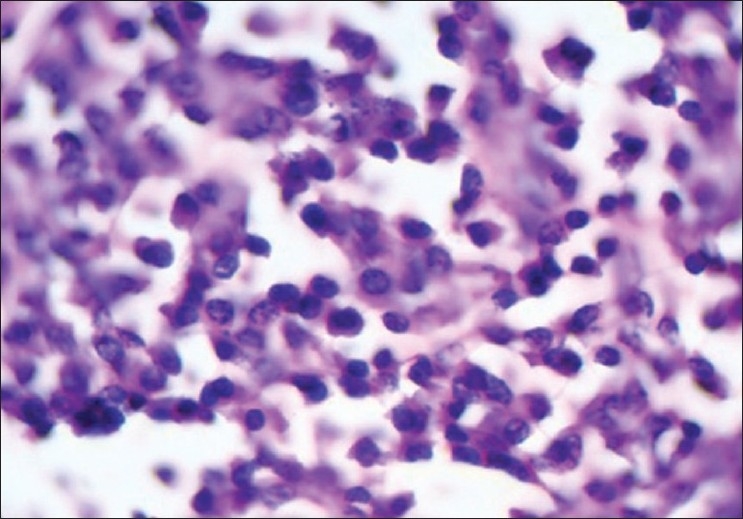
Plasmacytoma. Oil immersion Microwave processed

**Figure 11:**
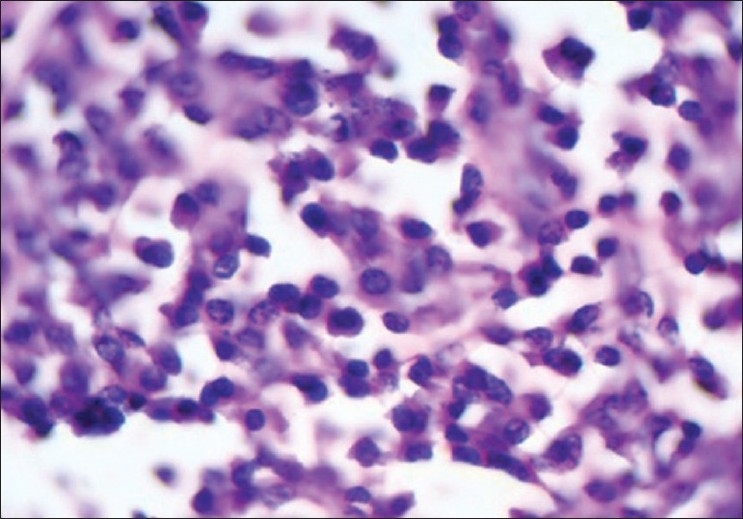
Plasmacytoma. Oil immersion Conventional processed

The microwave processed slides of malignancy cases such as angiosarcoma [Figure 6], verrucous carcinoma [Figure 8], and plasmacytoma [Figure 10] showed hyperchromatism and pleomorphism of tumor cells, which were also seen in conventionally processed slides [Figures 7, 9, 11]. This finding was in accordance with those of Hopewood *et al*.,[15] who accepted that pathological diagnosis, including malignancy, can be given satisfactorily by seeing the microwave processed slides.

There was excellent staining of tumor cells as compared to similar sections processed with conventional technique [Figures 6–9]. Irregular nuclear membrane, prominent nucleoli, and mitotic figures in malignant lesions were also clearly evident in the microwave processed tissue sections [Figure 2], which was also seen in studies by Mathai,[13] which indicates good nuclear morphology.

**Figure 12:**
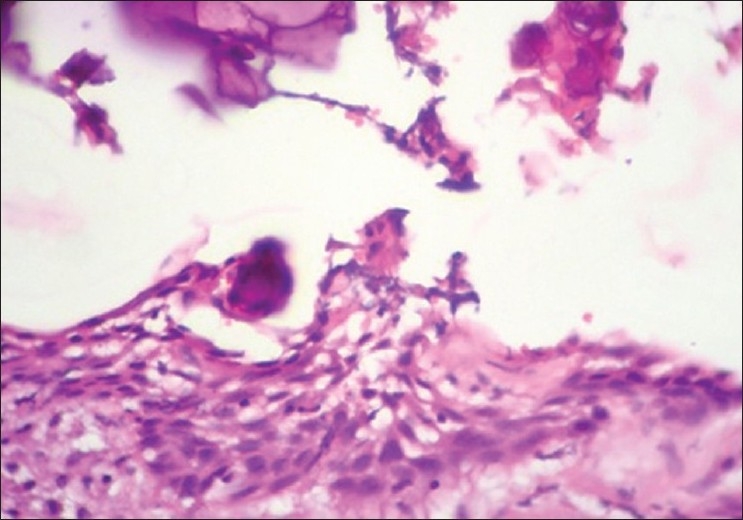
Calcifying epithelial odontogenic cyst. 10× Microwave processed

**Figure 13:**
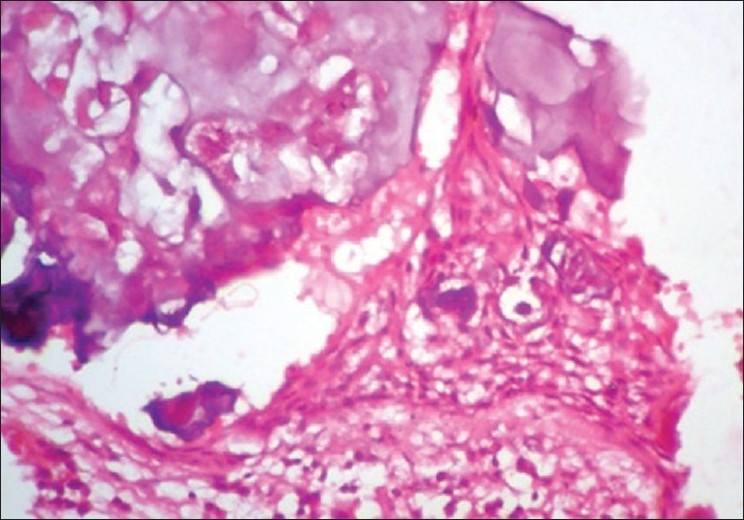
Calcifying epithelial odontogenic cyst. 10× Conventional processed

Foci of dystrophic calcification were preserved as seen in cases of Hopewood *et al*.,[15] and excellently seen in one case of calcifying epithelial odontogenic cyst in our study. Figures 12 and 13 show the cystic lumen with ghost cell calcification.

**Figure 14:**
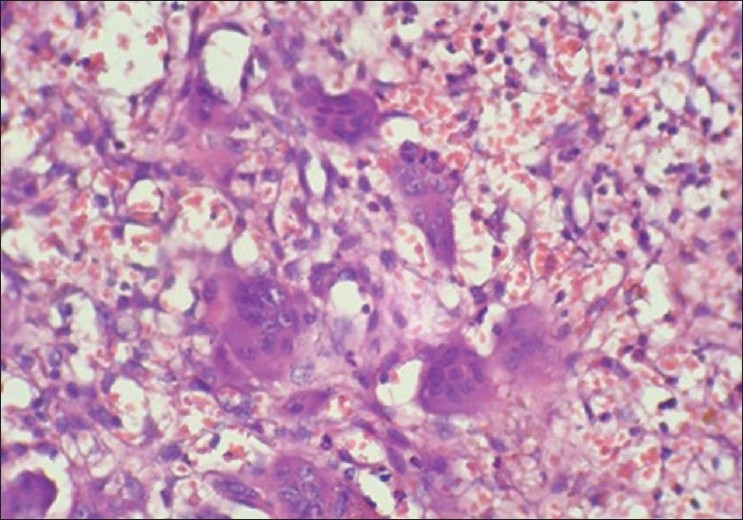
Central giant cell granuloma. 40× Microwave processed

**Figure 15:**
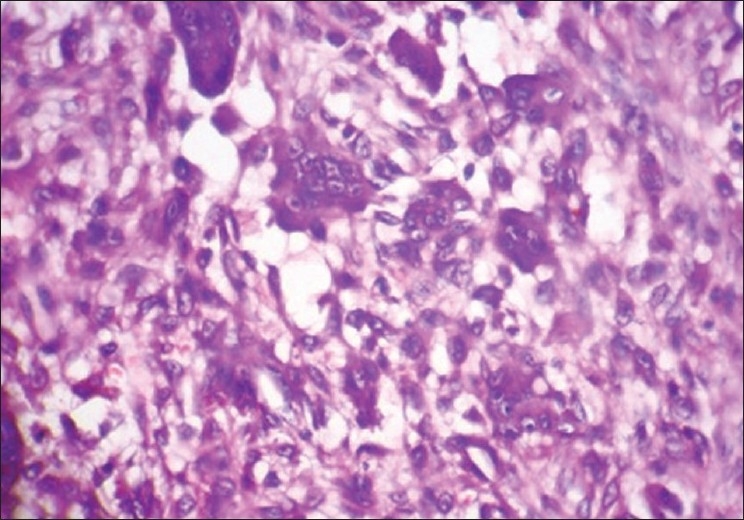
Central giant cell granuloma. 40× Conventional processed

There was no significant difference between nuclear size and shapes, as seen in [Figures 14 and 15] a case of central giant cell granuloma where the giant cells are showing 5-10 nuclei. The staining characteristics were discernable, as similarly seen in cases studied by Mathai.[13]

**Figure 16:**
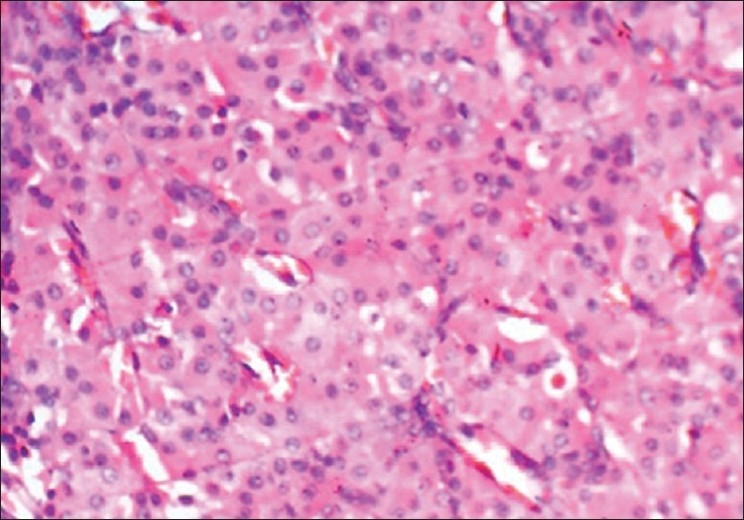
Oncocytoma. 40× Microwave processed

**Figure 17:**
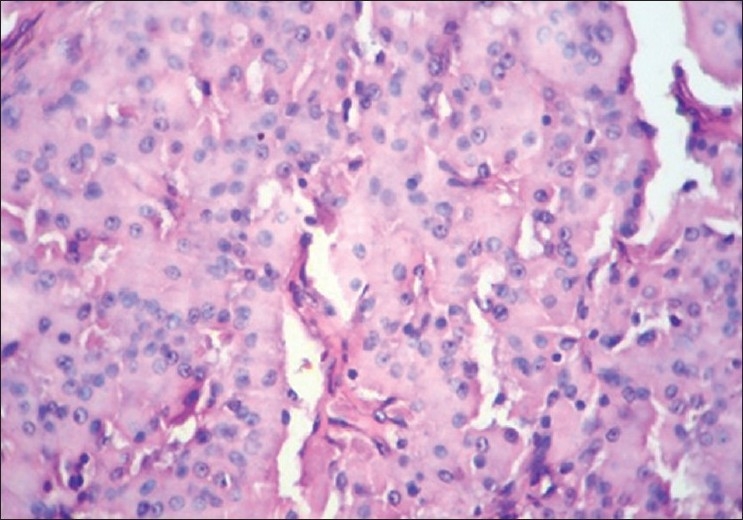
Oncocytoma. 40× Conventional processed

The staining of tissues in the microwave and conventionally processed slides did not show significant variation [Table 4, Figure 3]. This was in consonance with the findings of Boon *et al*.,[7] Chaudhari *et al*.,[5] Morales *et al*.,[11,12] Panja *et al*.,[14] Zenobia *et al*.,[18] Galvez *et al*.,[19] Suri *et al*.,[20] Leong *et al*.,[6,21] Kok *et al*.,[9] and Rohr *et al*.[22] Microwave processed tissue stained with hematoxylin and eosin were slightly more eosinophilic [Figures 16 and 17], with oncocytoma showing eosinophilic granular cytoplasm.

Similar findings were observed in the study carried out by Sivdas *et al*., where cytoplasm stained moderately eosinophilic, with no deeper eosinophilia observed.[13] Also, Hopewood *et al*.[15] and Leong[6] noted eosinophilia in tissues fixed by microwaves, independent of the solution in which the tissues were processed. He also mentions that this eosinophilia was readily corrected by altering the staining time in eosin. Eosinophilia of the cytoplasm also produced greater enhancement of the nuclear-cytoplasmic contrast, according to Leong.[4]

As the tissue that can be processed is very small, often the sample may not be representative of the site, and hence proper diagnosis could not be reached in few cases, which were in consonance with Suri *et al*.[20] This discrepancy was seen in both techniques.

Few sections seen in conventional technique showed artifacts, such as uneven staining. This was due to inadequate dehydration and also due to faulty clearing techniques during the staining process.

Moreover, residual paraffin in the tissue would have resulted in uneven staining.[23–25] Cellular morphology for the conventional technique was indistinct in 6%-20% [Figure 1] among all observers in this study; this was probably because of dehydration.[5] This was not seen in any of the studies so far. Even the staining quality of conventionally processed slides was satisfactory, and few cases showed poor staining quality as compared to microwave processed slides [Figure 3]. This could be explained by the fact that alcohols used in the processing lose their effectiveness as dehydrating agents, as they become diluted by moisture from the atmosphere and tissues. In the stained section, the inadequately dehydrated tissue gets partially unstained.[8]

Right from pre-cancerous conditions and lesions to malignancy, reactive lesions to benign tumors, microwave technique has shown a remarkable difference without losing the architecture and morphology of the cells.

## CONCLUSIONS

The slides obtained by microwave processing did not in any way alter the cellular morphology, nuclear morphology, or staining characteristics. A comparison of the microwave processed slides with the conventionally processed slides shows that the results are statistically insignificant.

In addition, the profitability of any diagnostic laboratory would be increased by using this technique, as this will enable handling a large batch of samples in a single day. Moreover, this will be a boon for the technical personnel whose work practices and lifestyles would change for the better, and this is something which defies statistical analysis.[6]
